# Fully automated image quality evaluation on patient CT: Multi-vendor and multi-reconstruction study

**DOI:** 10.1371/journal.pone.0271724

**Published:** 2022-07-20

**Authors:** Minsoo Chun, Jin Hwa Choi, Sihwan Kim, Chulkyun Ahn, Jong Hyo Kim

**Affiliations:** 1 Department of Radiation Oncology, Chung-Ang University Gwang Myeong Hospital, Gyeonggi-do, Republic of Korea; 2 Institute of Radiation Medicine, Seoul National University Medical Research Center, Seoul, Republic of Korea; 3 Department of Radiation Oncology, Chung-Ang University College of Medicine, Seoul, Republic of Korea; 4 Department of Applied Bioengineering, Graduate School of Convergence Science and Technology, Seoul National University, Seoul, Republic of Korea; 5 Department of Transdisciplinary Studies, Program in Biomedical Radiation Sciences, Graduate School of Convergence Science and Technology, Seoul National University, Seoul, Republic of Korea; 6 ClariPi Research, Seoul, Republic of Korea; 7 Department of Radiology, Seoul National University College of Medicine, Seoul, Republic of Korea; 8 Department of Radiology, Seoul National University Hospital, Seoul, Republic of Korea; 9 Center for Medical-IT Convergence Technology Research, Advanced Institutes of Convergence Technology, Suwon, Republic of Korea; University of Texas at Arlington, UNITED STATES

## Abstract

While the recent advancements of computed tomography (CT) technology have contributed in reducing radiation dose and image noise, an objective evaluation of image quality in patient scans has not yet been established. In this study, we present a patient-specific CT image quality evaluation method that includes fully automated measurements of noise level, structure sharpness, and alteration of structure. This study used the CT images of 120 patients from four different CT scanners reconstructed with three types of algorithm: filtered back projection (FBP), vendor-specific iterative reconstruction (IR), and a vendor-agnostic deep learning model (DLM, ClariCT.AI, ClariPi Inc.). The structure coherence feature (SCF) was used to divide an image into the homogeneous (***R***_***H***_) and structure edge (***R***_***S***_) regions, which in turn were used to localize the regions of interests (ROIs) for subsequent analysis of image quality indices. The noise level was calculated by averaging the standard deviations from five randomly selected ROIs on ***R***_***H***_, and the mean SCFs on ***R***_***S***_ was used to estimate the structure sharpness. The structure alteration was defined by the standard deviation ratio between ***R***_***S***_ and ***R***_***H***_ on the subtraction image between FBP and IR or DLM, in which lower structure alterations indicate successful noise reduction without degradation of structure details. The estimated structure sharpness showed a high correlation of 0.793 with manually measured edge slopes. Compared to FBP, IR and DLM showed 34.38% and 51.30% noise reduction, 2.87% and 0.59% lower structure sharpness, and 2.20% and -12.03% structure alteration, respectively, on an average. DLM showed statistically superior performance to IR in all three image quality metrics. This study is expected to contribute to enhance the CT protocol optimization process by allowing a high throughput and quantitative image quality evaluation during the introduction or adjustment of lower-dose CT protocol into routine practice.

## Introduction

Computed tomography (CT) is a well-established diagnostic imaging modality, with the number of CT scans continuously increasing for a variety of disease examinations and screening purposes [1]. Because of the potential risks of radiation-induced cancer from the associated radiation exposure [2,3], optimizing the scan protocols has become a crucial task in routine imaging practices that consider the patient’s weight and age as well as body parts being scanned [4]. The scan protocol optimization requires balancing the radiation dose with sufficient image quality such that the necessary diagnostic information is not compromised [4]. Last decade has seen the developments of numerous techniques for radiation dose reduction including automatic exposure control [5–7], dual-energy imaging [8,9], iterative reconstruction (IR) [7,10–12], and deep learning-based reconstruction (DLR) [13]. IR techniques have been highlighted over the last decade with their significant dose reduction performance along with image quality improvements [14–17], and are widely accepted in routine imaging practices [18–21]. Recently, novel DLR techniques have also been developed and become commercially available by CT manufacturers and third party developers: an advanced intelligent Clear-IQ Engine (AiCE, Canon Medical System, Otawara, Japan), a TrueFidelity CT (GE Healthcare, Milwaukee, WI), PixelShine (AlgoMedica Inc, Sunnyvale, CA), and ClariCT.AI (ClariPi Inc, Seoul, South Korea) [22–34].

Phantom-based approaches focused on the performance evaluation of the CT scanner by quantitatively assessing noise characteristics and lesion detectability. According to the evaluation tasks, a task-specific phantom is a preferable option rather than directly scanning human objects in spite of their oversimplified nature and lack of the variability present in patient population [35]. A newly-devised task transfer function analyzes the signal transfer characteristics across scanners, reconstruction algorithms, and imaging tasks, enabling to apply not only FBP but also IR and DLR [36,37]. However, even with use of same scanner, scan protocols, and reconstruction techniques, image qualities in patients can differ across them because of inter-patient variation and intrinsic patient-specific status. Naturally, appropriate links between phantom and patient images are necessary to characterize physical parameters to clinically relevant metrics [38,39]. One of the most frequently employed metrics is a noise magnitude, which is a primary basis for the image quality assessment task [40]. Most IR and DLR-related publications reported significant noise reduction, thus enabling to effectively reduce radiation doses [41–46]. However, several studies reported that the degradation of imaging resolution and altered image textures caused by IR is inevitable and this might compromise the visibility of small-scale and diagnostically important tissue structures [47–52].

A few studies attempted to evaluate the image quality of IR techniques in terms of sharpness degradation of anatomical structures of interest in a qualitative manner. Deák et al. performed subjective image quality assessment of abdominal CT images with model-based IR (MBIR, GE Healthcare) and reported its superior resolution and contour delineation of tissue interfaces as well as noise reduction as compared to filtered back projection (FBP) and adaptive statistical iterative reconstruction (ASIR, GE Healthcare) [53]. Another abdominal CT study compared profile tendencies for FBP and model-based IR images on both high-contrast features (an air hole) and moderate contrast from the edges of the liver to the agar region [54]. However, these approaches required manual drawing of contours which needs special care of operator to avoid confounding structures such as vessels and lesions [55]. These show the necessity of an objective image quality assessment method that is directly applicable to patient CT images and measure clinically relevant image quality metrics for different reconstruction techniques.

Previously, we presented a fully automated method for noise measurement from patient CT images by employing the structure coherence feature (SCF) which calculating the likelihood of pixels belonging to anatomical structures [56]. We applied a low threshold to the calculated SCF to enable an automated segmentation of homogeneous regions suitable for placing a region of interest (ROI) in the measurement of noise level. In this study, we extend our previous study to enable a novel image quality assessment method which extracts a set of image quality metrics such as structure sharpness and preservation of tissue structures as well as noise level from patient CT images by employing the same SCF. First, the reference organ (liver) was segmented by using a deep learning segmentation model. Then, a thresholding was applied with two cut-off values to the calculated SCF within the reference organ to further split the segmented region into homogenous and structural transition regions, from which we extract a set of image quality metrics in a fully automated way. We apply our novel image quality assessment method to patient image dataset of contrast-enhanced liver CT exams with CT scanners from 4 different manufacturers reconstructed with FBP, IR, and DLR techniques. We show that our proposed method could robustly extract image quality metrics regardless of CT manufacturer and reconstruction techniques.

## Materials and methods

### Dataset

An institutional review board at Seoul National University Hospital approved this retrospective study (IRB No. 1905−077−1033), and the informed consent was waived due to the retrospective design. A total of 120 patients’ contrast-enhanced liver CT scans, whose personal information tags in DICOM files were anonymized, were retrospectively collected. The CT datasets were acquired using four multi-detector CT scanners: Scanner 1 (GE Discovery CT750 HD, GE Healthcare, Milwaukee, WI), Scanner 2 (Ingenuity CT, Philips Healthcare, Cleveland, OH), Scanner 3 (SOMATOM Definition Flash, Siemens Healthcare, Erlangen, Germany), and Scanner 4 (Aquilion ONE, Canon Medical Systems, Otawara, Japan). Images of 30 patients per scanner were collected, and all scans were performed using automatic exposure control (AEC). Detailed parameters are summarized in Table 1.

**Table 1:** Acquisition parameters of CT scans.

	Scanner 1	Scanner 2	Scanner 3	Scanner 4
Number of cases	30	30	30	30
Tube voltage (kV)	120	100	100	100
Mean tube current (mAs)	131.5 ± 27.3	153.2 ± 25.8	151.7 ± 29.3	151.3 ±32.2
CTDI_vol_^a^ (mGy)	10.4 ± 2.1	7.2 ± 1.1	6.1 ± 1.3	7.9 ± 1.7
DLP^b^ (mGy cm)	541.9 ± 141.7	384.9 ± 69.7	283.4 ± 73.4	404.7 ± 93.3
Slice thickness (mm)	2.5 mm	3 mm	3 mm	3 mm

^a^CTDI_vol_ = Volumetric CT dose index.

^b^DLP = dose-length product.

Three types of reconstruction techniques were utilized: conventional FBP, vendor-specific IRs, and a vendor-agnostic deep learning model (DLM) (ClariCT.AI, ClariPi Inc., South Korea). Vendor-specific IRs were as follows: ASIR (Adaptive statistical iterative reconstruction, GE Healthcare), iDose (Philips Healthcare), SAFIRE (Sinogram affirmed iterative reconstruction, Siemens Healthcare), and AIDR3D (adaptive iterative dose reduction 3D, Canon Medical Systems) [41–45]. Details about ClariCT.AI are described in below subsection.

#### DLM (ClariCT.AI)

The DLM (ClariPi Inc., South Korea) [57] was established using a convolutional neural network (CNN) algorithm and enabled image denoising by training with the paired set of simulated low-dose and original CT images as input and output, respectively [32–34,58]. To acquire a generalized learning and vendor-agnostic denoising capability, the dataset consisted of over 1 million CT images encompassing 2,100 different combinations of scan and reconstruction conditions, including varying kV, mAs, automatic exposure control, slice thickness, contrast enhancement, and convolution kernels with 24 scanner models from four different CT manufacturers. The clarity-weighted option was employed, which produced denoised images with sharpness enhancement to a degree tuned to compensate for the image blurring effect. The performance of DLM has previously been evaluated in several clinical studies [16,31–34,59–61].

### Reference organ segmentation

**Fig 1:**
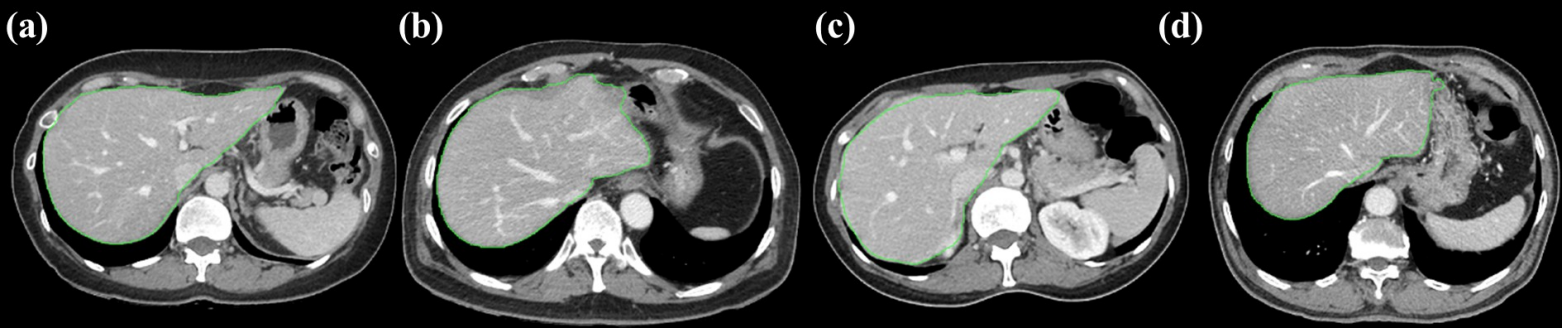
Sample liver segmentation results generated by the deep learning segmentation model. The results were displayed for multi-vendor CT images acquired through (a) Scanner 1, (b) Scanner 2, (c) Scanner 3, and (d) Scanner 4, respectively.

This study used liver as the reference organ for image quality assessment [62]. A deep learning segmentation model was trained using a U-net architecture and 257 CT scans [63]. The ground truth liver mask was established by a radiation oncologist having two years of experience. The deep learning segmentation model was optimized using adaptive momentum estimation (Adam), and an early stopping technique was used to prevent overfitting [64,65]. The established model was validated using 110 CT scans and exhibited 94.5±16.8% of dice similarity coefficient with the ground truth [66]. Fig 1 shows sample segmentation results for the image dataset. From the entire images, those with segmented areas greater than 100 cm^2^ were selected for further process of image quality evaluation.

### SCF

The SCF was calculated for each pixel within the segmented reference organ. The SCF consisted of an edginess feature and directional entropy feature. The edginess feature represented the likelihood of a pixel being located on an anatomical structure, whereas the directional entropy feature represented the randomness of the pixel orientation to signify the absence of an anatomical structure [56]. The SCF is defined as follows:

$$SCF=\frac{\sum_{(i,j)\in ROI}I_{E}(i,j)}{H_{G}+H_{T}}$$ (1)

where ***I***_***E***_, ***H***_***G***_, and ***H***_***T***_ denote the edginess of each pixel, directional entropy for the gradient vector, and structure tensor, respectively [56]. As shown in Eq (2), the edginess was introduced to present transitional edge between distinct region, and defined by the weighted sum of the magnitude for the gradient (|∇*I*|) and the 1^st^ eigenvalue of structure tensor (|*λ*_1_|),

$$I_{E}=\omega_{1}|\nabla I|+\omega_{2}|\lambda_{1}|$$ (2)

and *ω*_1_ and *ω*_2_ are the corresponding weight. The sum of directional entropies for the gradient vector and structure tensor (***H***_***G***_, ***H***_***T***_) are employed to describe the curvilinear edge caused by tubular structures. Directional entropies (***H***_***G***_, ***H***_***T***_) are calculated by below formulas [56,67]:

$$H_{G}=-\sum_{(i,j)\in ROI}P(∠\nabla \;I)\ln\;P(∠\nabla \;I)$$ (3)

$$H_{T}=-\sum_{(i,j)\in ROI}P(∠V_{T})\ln\;P(∠V_{T})$$ (4)

where ***V***_***T***_ was the 1st eigenvector of structure tensor ***T***, which is defined as,

$$T=\left|\begin{array}{cc} {I_{x}}^{2} & I_{x}I_{y}\\ I_{x}I_{y} & {I_{y}}^{2} \end{array}\right|=\left|\begin{array}{cc} T_{11} & T_{12}\\ T_{12} & T_{22} \end{array}\right|$$ (5)

$$V_{T}=\left(\begin{array}{c} T_{22}-T_{11}+\sqrt{(T_{22}-T_{11}{)}^{2}+4{T_{12}}^{2}}\\ -2T_{12} \end{array}\right)$$ (6)

### Extraction of image quality metrics

#### Noise level estimation

A thresholding was applied to the calculated SCF with the cut-off point at the 10^th^ percentile, which resulted in the segmentation of homogeneous regions (***R***_***H***_). Then, five ROIs were randomly selected within ***R***_***H***_, from which the average standard deviation of pixel values was set as the noise level.

*Validation of noise level measurement*. To demonstrate the reliability between automated and manual noise level measurements, a single FBP image from 120 patients’ images (total 120 images) were used. An experienced radiologist manually drawn ROIs on uniform soft tissue area, and obtained noise levels by taking standard deviation. The Pearson correlation coefficient was calculated to evaluate the agreement between the manual and automated measurements. Furthermore, Bland-Altman plots were drawn to demonstrate the mean difference and systematic bias.

#### Structure sharpness index (SSI)

**Fig 2:**
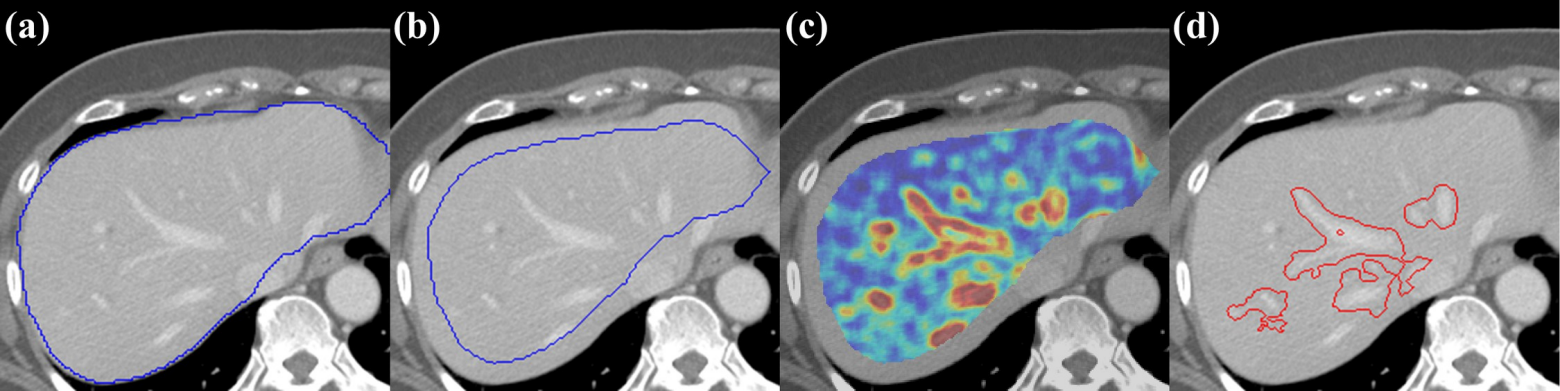
(a) A sample image of which the image quality being assessed with candidate region (liver parenchyma) segmented by using U-net deep-learning model, (b) Eroded mask from candidates with a structural element of 7 pixels, (c) SCF heat map calculated on the eroded mask, and (d) structural transition regions where the edge sharpness being quantified.

**Fig 3:**
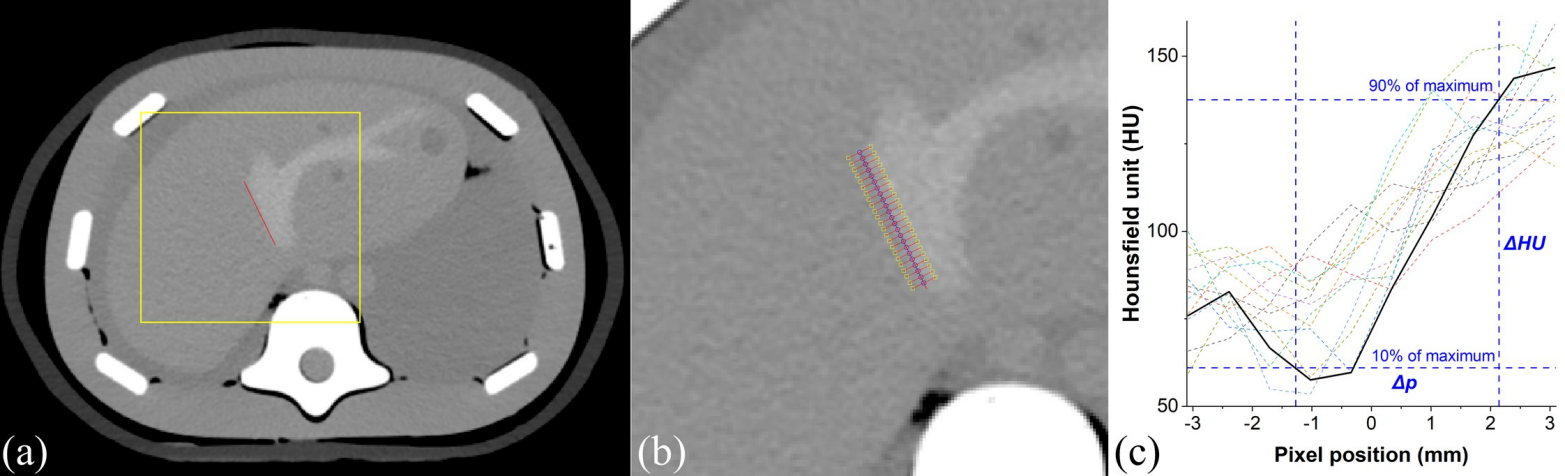
(a) An example showing enhanced vessel on a triple modality 3D abdominal phantom where the red line indicates the manually drawn edge component. (b) Perpendicular line profiles on given manual edge line. (c) Dashed lines show the edge rise trend signifying the vessel sharpness, whereas the bold solid line denotes the average value.

The structure transition regions between two different tissues were extracted by applying a high SCF threshold. We empirically observed that the 70^th^ percentile of SCF was appropriate for localizing enhanced hepatic regions, and the region was denoted as ***R***_***S***_ (Fig 2(D)). Regions with SCF from the 10^th^ to 70^th^ percentiles did not belong to any specified region. The sharpness surrogate was then defined as the mean of the SCF within the segmented ***R***_***S***_. In order to establish the relationship between manual and SCF-based automated slope measurement, a triple modality 3D abdominal phantom (CIRS Inc., Norfolk, VA) was scanned using Brilliance Big Bore CT (Philips, Amsterdam, The Netherlands) to verify if the SSI measured as above appropriately represent vessel sharpness in the contrast enhanced liver CT. The scans were performed at 120 kV, 300 mAs, and reconstructed with filtered back projection and iDose3 using three different kernels (smooth, standard, sharp) each. The six images were intentionally smoothed by varying Gaussian sigma from 0.5 to 1.6, and sharpened with an unsharp mask by varying the amount parameter from 1.5 to 2.5 [68], and eventually 264 images were arranged. For each dataset, the edge line between the normal tissue and enhanced vessel was manually drawn, and their corresponding perpendicular line profiles were obtained (Fig 3B) [69] along the edge line at equal distance. The mean slope of the perpendicular line profile was used as a reference sharpness measure of the enhanced vessel. The 90% and 10% of maximum intensities and their pixel positions on profiles were utilized [69] to calculate the sharpness (*ξ*_*l*_) of the line profiles using Eq (7),

$$\xi_{l}=\frac{\mathrm{cov}[s_{l},I_{l}]}{\mathrm{var}[s_{l}]}=\frac{\sum_{i}\left(s_{l,i}-\frac{1}{N_{l}}\sum_{j}s_{l,j})(I_{l,i}-\frac{1}{N_{l}}\sum_{j}I_{l,j}\right)}{\sum_{i}{\left(s_{l,i}-\frac{1}{N_{l}}\sum_{j}s_{l,j}\right)}^{2}}$$ (7)

where ***s***_*l*_ contains the distances in physical units of the *N*_*l*_ sample locations within the region of interest, and ***I***_*l*_ denotes the corresponding intensities along the profile line *l* [69]. A linear regression was performed to characterize the relationship between the manual and the SCF-based automated sharpness measurement yielding a relationship,

$$\xi_{l}=6.1398\times {\bar{f}}_{s}+4.2813$$ (8)

*Validation of SSI measurement*. To demonstrate the reliability between estimation and manual *ξ*_*l*_ measurements, a single image from 120 patients’ images (total 120 images) were used, and the enhanced vessel line was manually drawn by an experienced radiologist. The *ξ*_*l*_ were measured manually and automatically by using Eqs (7) and (8), respectively. Same as a validation on noise level measurement, the Pearson correlation coefficient and Bland-Altman plot were drawn to present the agreement, and limits of agreement, respectively.

#### Structure alteration index (SAI)

Subtraction between two CT images is an effective way to assess the structure alteration caused by the IR algorithm, which is difficult to recognize by visual analysis without subtraction [70]. We devised a novel structure alteration index that conveniently quantify the degree of structure alteration. By employing our ROI extraction scheme for structure and homogenous regions, we extracted the pixel standard deviation within the structure and homogenous ROIs and derived their ratio as follows:

$$SAI={\frac{\sigma_{R_{S}}}{\sigma_{R_{H}}}|}_{\Delta I}$$ (9)

where σ denotes the standard deviation, the ***R***_***S***_ and ***R***_***H***_ denote the structure and homogenous ROIs being evaluated, respectively with ***ΔI*** indicating the subtraction between the reference (FBP) and target (IR or DLM) images. Therefore, the SAI value will be lower when the target reconstruction algorithm reduces the noise without significant alteration of the structural components. In contrast, the SAI will increase if substantial residual structures exist in the subtraction image domain.

## Results

### Noise level assessment

**Fig 4:**
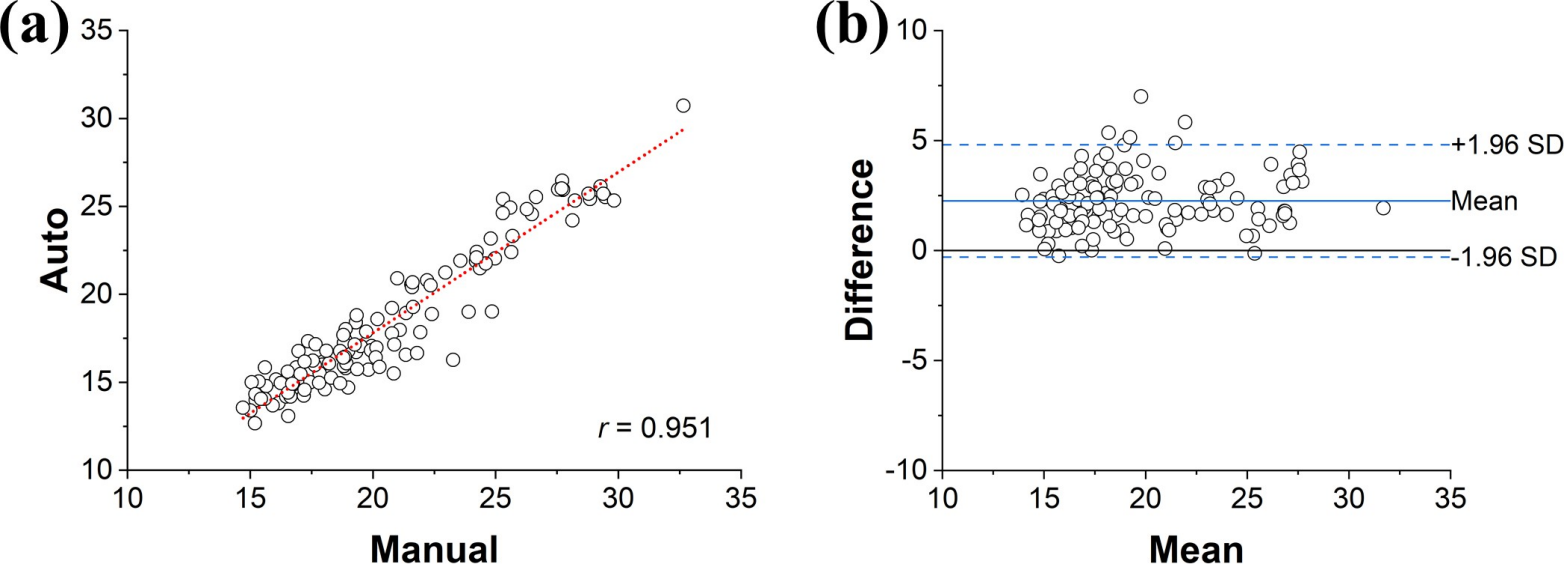
Agreements between automated and manual noise level measurement. (a) A scatter plot with linear regression showing a Pearson’s correlation coefficient of 0.951, and (b) a Bland-Altman plot where the solid and dashed red lines indicate the mean difference and 95% limits of agreement.

As shown in Fig 4, automated noise level measurements showed a desirable reliability with manual measurement by showing Pearson’s correlation coefficient of 0.951, mean difference of 2.26, standard deviation of 1.30, and 95% limits of agreement of [-0.29, 2.26].

**Fig 5:**
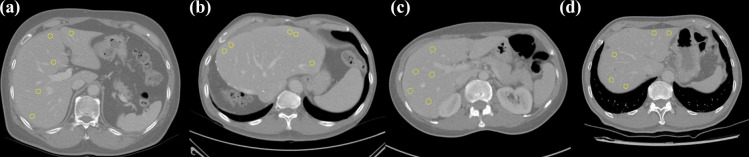
Sample homogeneous ROI extraction results on contrast-enhanced liver CT exams. The results were displayed for multi-vendor CT images acquired through (a) Scanner 1, (b) Scanner 2, (c) Scanner 3, and (d) Scanner 4, respectively.

**Fig 6:**
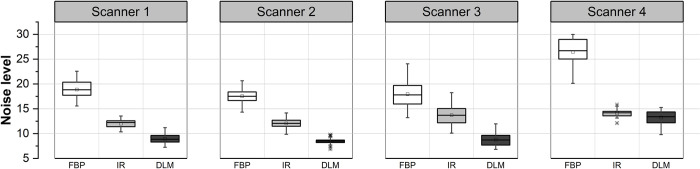
Box plots compare the noise level measurements among the multi-vendor and multi-reconstruction images.

A total of 722, 923, 873, and 682 images were used for image quality evaluation of Scanners 1, 2, 3, and 4, respectively. Several samples for localizing homogeneous ROIs using fully automated methods are presented in Fig 5. The validation of an automated measurement of noise levels by comparing those with manual ROI placement was reported in our earlier study [56], and thus skipped in this study. The automated noise levels across FBP, IR, and DLM are compared in Fig 6. Compared with conventional FBP reconstruction, mean noise levels decreased by 35.9%, 31.2%, 23.5%, and 46.7% with iterative reconstruction and 52.6%, 51.6%, 51.1%, and 50.0% with ClariCT.AI for Scanners 1, 2, 3, and 4, respectively. The magnitudes of noise levels were in the order of FBP, IR, and DLM, with statistically significant differences (*p* < 0.001).

### Structure sharpness index

**Fig 7:**
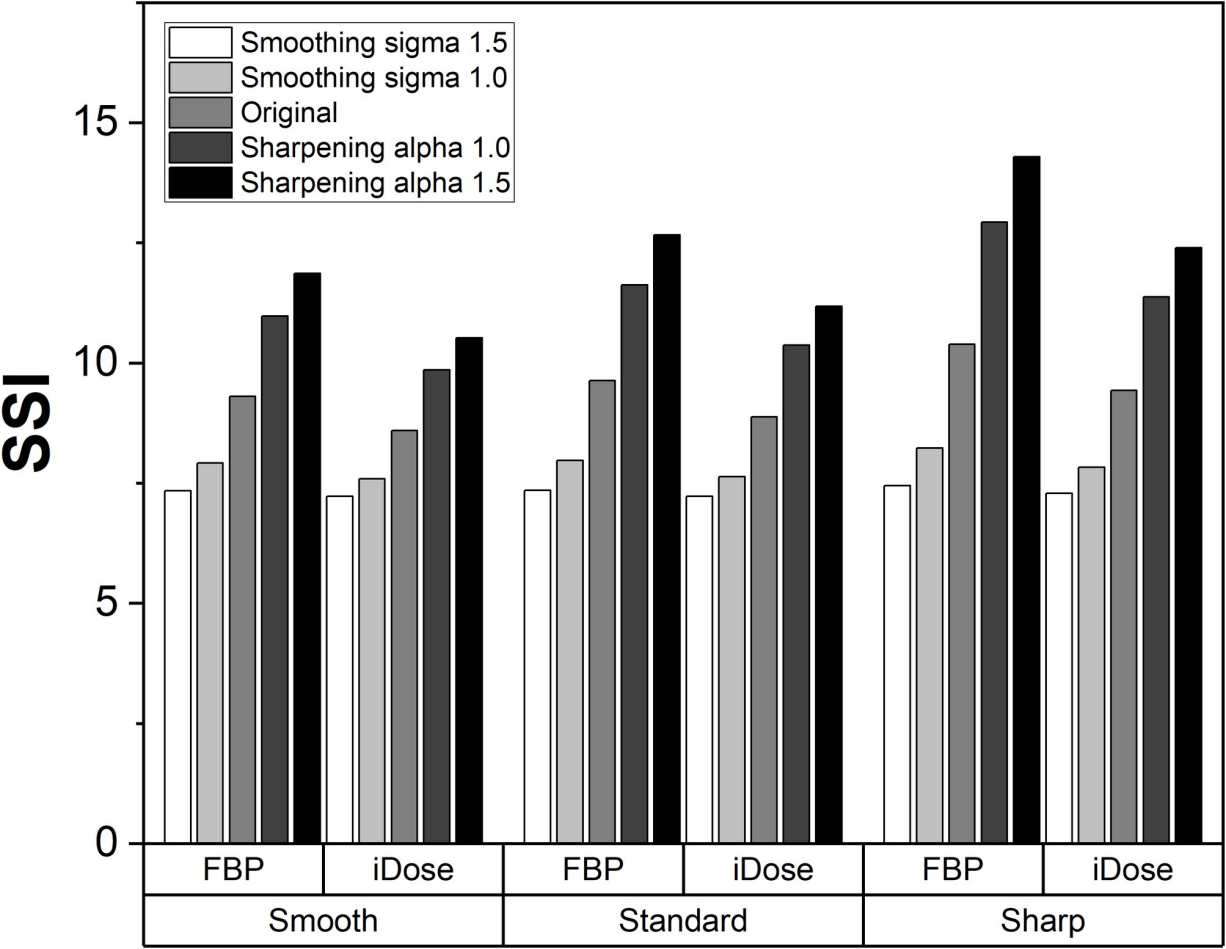
SSI comparison acquired at a triple modality 3D abdominal phantom data. The data included reconstructions with smooth, standard, and sharp kernel along with two kinds of reconstruction methods (FBP and IR). The images with artificially manufactured of smoothing and sharpening were included.

Fig 7 showed SSIs from a triple modality 3D abdominal phantom data, which presents dependencies on reconstruction kernels and methods. In original FBP data (no smoothing or sharpening), images with smooth and sharp kernels showed -3.44% and 7.80% SSI changes, respectively, compared to those with standard kernel. Furthermore, those with iDose showed -7.86% SSI changes compared to those with FBP in standard kernel.

**Fig 8:**
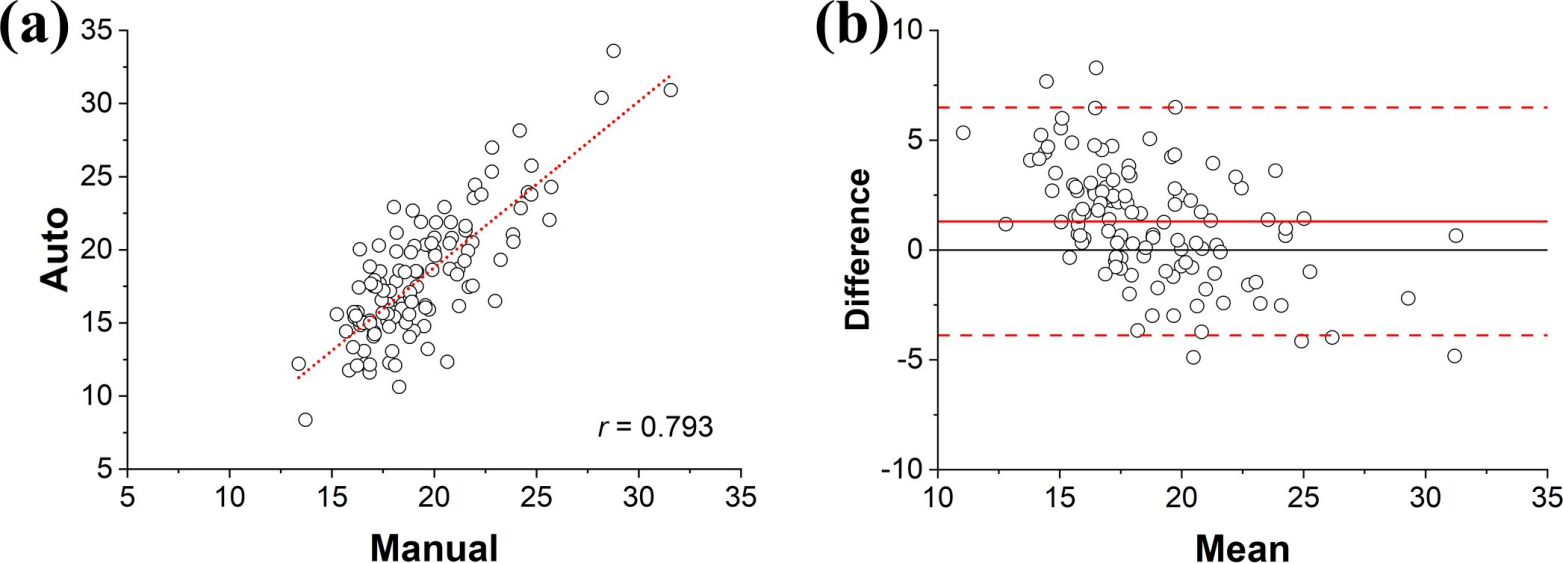
Agreements between automated and manual sharpness measurement. (a) A scatter plot with linear regression showing a Pearson’s correlation coefficient of 0.793, and (b) a Bland-Altman plot where the solid and dashed red lines indicate the mean difference and 95% limits of agreement.

As shown in Fig 8, an application to the 120 patients’ images presented the reliabilities between estimation and manual *ξ*_*l*_ measurements with Pearson’s correlation coefficient of 0.793, mean difference of 1.31, standard deviation of 2.65, and 95% limits of agreement of [-3.88, 6.49].

**Fig 9:**
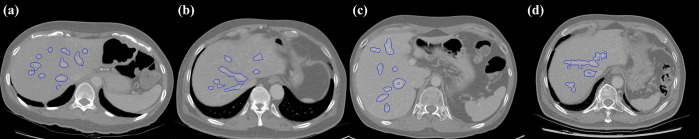
Sample localization results of structural transition region on enhanced hepatic region. The results were displayed for multi-vendor CT images acquired with (a) Scanner 1, (b) Scanner 2, (c) Scanner 3, and (d) Scanner 4, respectively.

**Fig 10:**
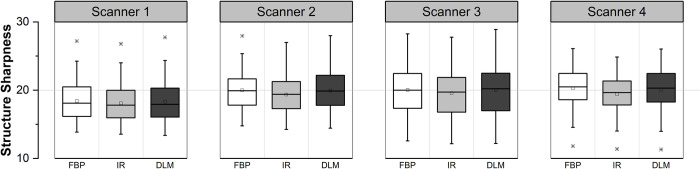
Box plots compare the structure sharpness index across multi-vendor and multi-reconstruction images.

By thresholding the SCFs with 70^th^ percentile, it was possible to discriminate the parenchyma-vessel transition region on enhanced hepatic region. Example localization results for the CT images with different manufacturers are shown in Fig 9. Measurements of SSI across the FBP, IR, and DLM datasets are shown Fig 10. Compared to conventional FBP reconstruction, mean sharpness decreased by 1.72%, 3.21%, 2.36%, and 4.21% with iterative reconstruction and 0.57%, 0.39%, 0.23%, and 1.19% with DLM for Scanners 1, 2, 3, and 4, respectively. When data from all manufacturers were pooled, the SSI of DLM was 2.57% higher than that of IR, with statistical significance (*p* < 0.05).

### Structure alteration index

**Fig 11:**
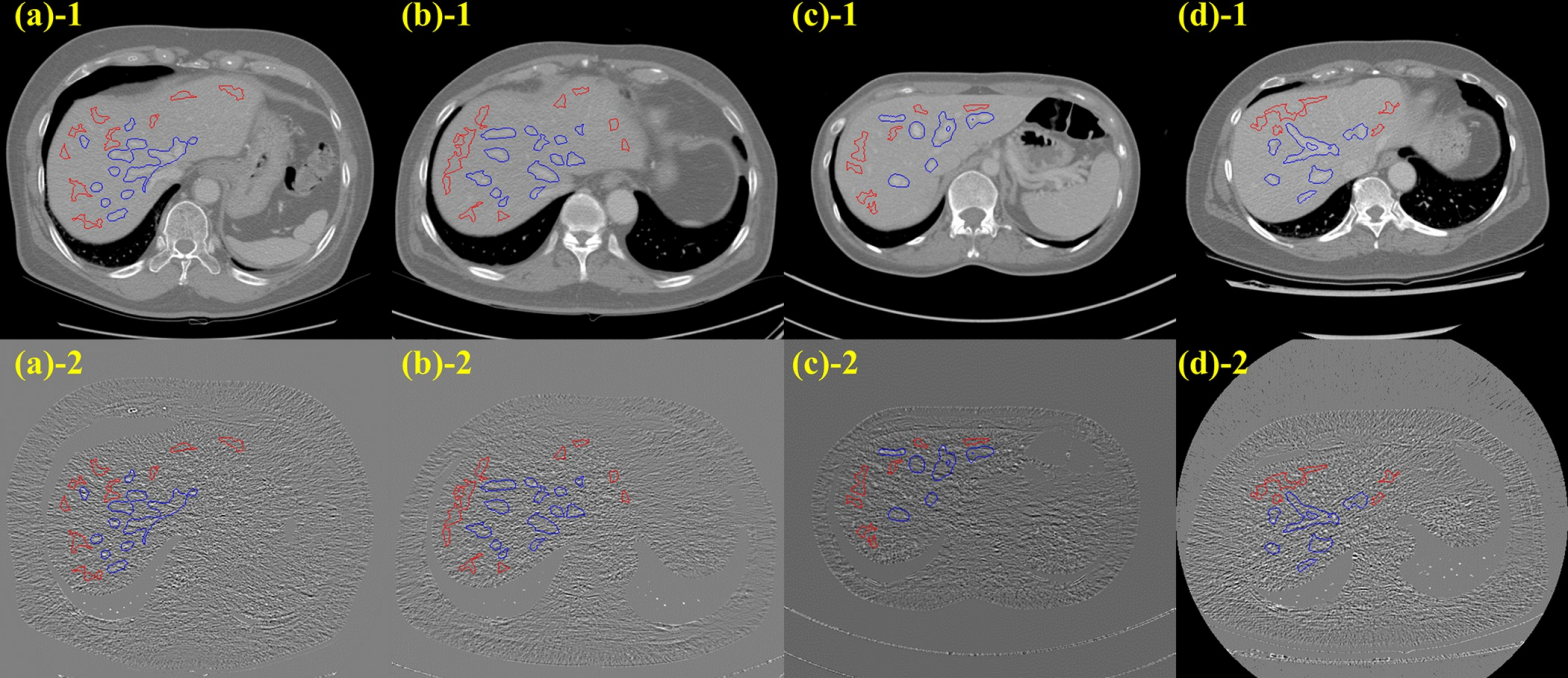
Upper row showed the homogeneous (red) and structure edge (blue) region on enhanced hepatic area displayed in the image domain, whereas the lower row shows same ROIs displayed on the subtraction image domain. Each image was acquired with a) Scanner 1, (b) Scanner 2, (c) Scanner 3, and (d) Scanner 4, respectively.

**Fig 12:**
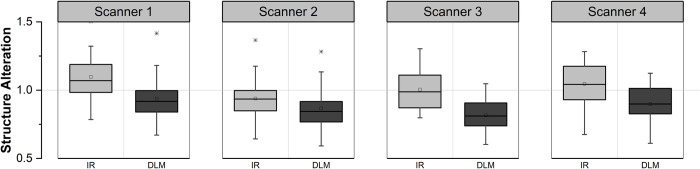
Box plots compare structure alteration index of IR and DLM images.

Sample localization results of homogeneous and structure edge regions on the subtraction image domain are shown in Fig 11, where subtraction can be either FBP and IR, or FBP and DL. Measurements of SAI across the FBP, IR, and DLM datasets are shown Fig 12. The mean SAI was 1.02 ± 0.16 and 0.88 ± 0.14 for IR and DLM, respectively. The difference of SAI between IR and DLM was statistically significant (*p* < 0.05).

## Discussion

In this study, we presented a novel image quality evaluation method that allows a fully automated assessment of three image quality metrics on patient CT images. We employed the structure coherence feature to enable an automated localization of homogeneous and structure edge regions on patient CT images from which the three key image quality metrics were calculated such as noise level, structure sharpness, and structure alteration [71,72]. We applied this method to the contrast enhanced liver CT images of 120 patients from four different CT scanners reconstructed with FBP, IR, and DLM.

Our study results showed the robust performance of the proposed method with successful extraction of image quality metrics for the patient image set with a variety of CT image quality. The extracted image quality metrics revealed the differences in the image quality characteristics of FBP, IR, and DLM. Overall, the measured noise level was in the order of FBP, IR, and DLM, whereas the noise reduction with IR and DLM was different depending on scanner manufacturers: DLM achieved 20–30% further noise reduction in three scanners but almost no further reduction in one scanner. Structure sharpness, which was difficult by visual assessment, was shown to degrade in IR but maintain in DLM compared to FBP. Furthermore, the newly introduced metric, SAI effectively quantified the changes in structure appearance after noise reduction, which was significantly higher in IR than in DLM across all the four CT manufacturers’ images.

To the knowledge of authors, this is the first study that reported the quantitative measurement of key image quality metrics in a fully automated way from patient CT images with objective comparison of image qualities among the FBP, IR, and DLM of different vendors. Frequently, noise levels were calculated as a standard deviation on uniform area, whose ROIs could be drawn manually or automatically [38,56,73–76]. Several studies attempted an automation by employing the global noise level approaches, which adopts the mode value in the histogram of pixel standard deviations on subtracted image domain across adjacent slices [38,73–75]. Their approaches were shown as robust by showing excellent correlation with reference values in abdominal, thoracic, and head CT scans. Another publication proposed an automated noise measurement technique by using an air-based global noise index in subtraction between adjacent images [76]. This technique is definitely beneficial due to their excellent correlation with those from patients as well as their global efficacy regardless of phantom or human images. They eliminated the needs to discriminate homogeneous regions in patient’s anatomy because air signal is inherently devoid with any anatomical structures. Our approach is not in accordance with previous ones by employing SCF, which intends to directly analyze how much ROIs contained structural transitions or not. As avoiding small scale-anatomical structure is also possible by adopting ROIs with low SCF values, noise levels obtained with automated measurements showed lower values than those with manual measurement. This infers SCF enable to select homogeneous ROIs with fully automated fashion, which is even hard for human observer (Fig 4(B)). Furthermore, ours enable to differentiate not only homogeneous area but also structural transition regions by using statistics of SCF, thus making it possible to evaluate noise level, SSI, and SAI with a fully automated approach.

Previous studies asserted that other aspects of image quality are also important and needed to be quantified in order to inspect the potential loss or degradation of diagnostically important image features such as visibility of small-scale structures. In fact, a study reported that one of the radiologists failed to detect small metastases on IR images owing to mild blurring and pixelation [77]. Therefore, we attempted to measure not only image noise, but also other important metrics that can influence the visual perception task in imaging diagnosis. We included structure sharpness and structure alteration in our triple metric image quality evaluation. Our results validated that the automated sharpness measurements presented in this study could reliably represent the manually measured structure slopes by an experienced observer with a strong Pearson correlation of 0.79. We devised the SAI in an attempt to inspect and report the subtle degradation of structure appearance caused by blurring or pixelation which was frequently reported to appear in IR. Evaluating the ability of SAI to report such structure degradation in newly introduced denoising techniques would be an interesting study, but was outside the scope of our study and thus remains a subject of further study.

In SSI measurements obtained with triple modality phantom data (Fig 7), they exhibited reliable representations of the sharpness by showing higher SSI in sharpened with greater alpha whereas lower in severely smoothed data (Gaussian sigma 1.5). Furthermore, it is noticeable that SSIs increased from smooth to sharp kernel, and decreased from FBP to iDose in same manipulation data. Although there existed no significant changes in patients’ SSI measurement across reconstruction methods (Fig 10), they are also meaningful because the sharpness of the enhanced-vessel structures well preserved while remarkably reducing the noise level.

This study had several limitations. Firstly, although we evaluated a vendor-agnostic DLM along with vendor-specific IRs in this study, other vendor-specific DLM algorithms such as AiCE (Canon Medical System) and TrueFidelity CT (GE Healthcare) were not included. Therefore, our study results for vendor-agnostic DLM cannot be generalized. We included the patient CT images of contrast enhance liver scanned with regular radiation dose settings. As the newly developed denoising techniques could find their full merits in application to low-dose CT (LDCT) examinations such as lung cancer CT screenings, future studies will need to include the LDCT data for lung cancer screening with multiple types of reconstruction [78–80]. In the LDCT lung cancer screening program, we hope the use of noise level, SSI, and SAI metrics could quantify image qualities, and provide insights to adopt best scan and reconstruction protocols. Our image quality metrics did not evaluate the image texture appearance. The changes in noise texture, including the grain size and shape of the noise and cartoon-like appearance in several IR algorithms will eventually lead to a shift in the peak frequency of the NPS [81]. Even with a similar amount of noise level, the observers’ diagnostic performance can also be influenced by noise texture alteration [82–85].

Despite these limitations, our study is expected to contribute to enhance the CT protocol optimization process by allowing a high throughput and quantitative image quality evaluation during the introduction or adjustment of lower-dose CT protocol into routine practice. Currently, the concept of diagnostic reference level in CT is moving to the noise and dose reference levels, which emphasizes the importance of not only radiation dose but also image quality in the justification of CT scanning according to the ALARA principle [86,87]. Therefore, our proposed method might contribute to integrate the image quality and radiation dose monitoring into quality assurance practice during CT scanning protocol optimization procedure.

## Conclusions

We have developed a fully automated method for image quality evaluation on patient CT images using three image quality metrics such as noise level, structure sharpness index, and structure alteration index. We demonstrated the robust performance of our proposed method with the patient images of contrast-enhanced liver CT from four different scanners reconstructed using FBP, IR, and DLM. Our proposed method has a potential to enhance the CT protocol optimization process by allowing a high throughput and quantitative image quality evaluation for not only phantom tests but also patient scan datasets.

## Supporting information

S1 Table Image quality analysis results for 120 patients. The noise levels, structure sharpness index, and structure alteration index are provided on average.
(DOCX)
