# Supplementary material for: Fully automated image quality evaluation on patient CT: Multi-vendor and multi-reconstruction study
Source: PLoS One. 2022 Jul 20;17(7):e0271724. doi: 10.1371/journal.pone.0271724 (PMC9299323; doi:10.1371/journal.pone.0271724)
Supplement: S1 Table — The noise levels, structure sharpness index, and structure alteration index are provided on average. (DOCX) [file pone.0271724.s001.docx]

| **Patient #** | **Manufactuer** | **ManufacturerModelName** | **# of Images analyzed** | **Mean noise level** | | | **Mean sharpness** | | | **Preservation** | |
| --- | --- | --- | --- | --- | --- | --- | --- | --- | --- | --- | --- |
|  |  |  |  | **FBP** | **IR** | **DL** | **FBP** | **IR** | **DL** | **IR** | **DL** |
| 1 | GE MEDICAL SYSTEMS | Discovery CT750 HD | 28 | 22.570 | 13.546 | 11.033 | 21.662 | 21.153 | 21.549 | 1.225 | 1.043 |
| 2 | GE MEDICAL SYSTEMS | Discovery CT750 HD | 23 | 18.881 | 12.306 | 8.805 | 20.273 | 19.993 | 20.306 | 0.921 | 0.805 |
| 3 | GE MEDICAL SYSTEMS | Discovery CT750 HD | 30 | 18.509 | 11.406 | 8.585 | 13.861 | 13.554 | 13.371 | 1.217 | 1.118 |
| 4 | GE MEDICAL SYSTEMS | Discovery CT750 HD | 28 | 20.891 | 13.432 | 10.134 | 16.680 | 16.224 | 15.943 | 0.992 | 0.919 |
| 5 | GE MEDICAL SYSTEMS | Discovery CT750 HD | 25 | 18.518 | 11.640 | 8.465 | 19.516 | 19.198 | 19.575 | 1.022 | 0.846 |
| 6 | GE MEDICAL SYSTEMS | Discovery CT750 HD | 25 | 18.321 | 12.309 | 8.927 | 24.257 | 24.011 | 24.352 | 0.919 | 0.783 |
| 7 | GE MEDICAL SYSTEMS | Discovery CT750 HD | 24 | 20.674 | 12.544 | 9.628 | 16.459 | 16.110 | 16.160 | 1.116 | 0.976 |
| 8 | GE MEDICAL SYSTEMS | Discovery CT750 HD | 48 | 18.357 | 10.625 | 8.647 | 20.733 | 19.662 | 19.620 | 1.575 | 1.416 |
| 9 | GE MEDICAL SYSTEMS | Discovery CT750 HD | 25 | 16.903 | 10.737 | 7.718 | 16.038 | 15.793 | 16.062 | 1.079 | 0.903 |
| 10 | GE MEDICAL SYSTEMS | Discovery CT750 HD | 17 | 17.438 | 11.695 | 8.089 | 15.040 | 14.839 | 14.975 | 0.785 | 0.671 |
| 11 | GE MEDICAL SYSTEMS | Discovery CT750 HD | 24 | 20.063 | 13.065 | 9.793 | 20.716 | 20.118 | 20.333 | 1.060 | 0.941 |
| 12 | GE MEDICAL SYSTEMS | Discovery CT750 HD | 28 | 17.851 | 11.745 | 8.601 | 18.138 | 17.674 | 17.857 | 0.983 | 0.836 |
| 13 | GE MEDICAL SYSTEMS | Discovery CT750 HD | 45 | 21.736 | 13.163 | 10.831 | 27.197 | 26.798 | 27.754 | 1.248 | 1.026 |
| 14 | GE MEDICAL SYSTEMS | Discovery CT750 HD | 17 | 17.103 | 11.208 | 7.763 | 17.163 | 17.029 | 17.177 | 0.975 | 0.837 |
| 15 | GE MEDICAL SYSTEMS | Discovery CT750 HD | 22 | 19.615 | 12.630 | 9.205 | 18.199 | 17.934 | 18.366 | 1.030 | 0.840 |
| 16 | GE MEDICAL SYSTEMS | Discovery CT750 HD | 16 | 18.353 | 12.199 | 8.733 | 17.537 | 17.239 | 17.405 | 1.163 | 0.981 |
| 17 | GE MEDICAL SYSTEMS | Discovery CT750 HD | 24 | 15.743 | 10.357 | 7.638 | 16.154 | 15.952 | 16.065 | 1.057 | 0.892 |
| 18 | GE MEDICAL SYSTEMS | Discovery CT750 HD | 25 | 19.642 | 12.178 | 9.151 | 20.499 | 20.265 | 20.662 | 1.098 | 0.951 |
| 19 | GE MEDICAL SYSTEMS | Discovery CT750 HD | 16 | 19.132 | 12.676 | 9.053 | 14.949 | 14.614 | 14.697 | 1.001 | 0.886 |
| 20 | GE MEDICAL SYSTEMS | Discovery CT750 HD | 33 | 22.336 | 13.397 | 11.199 | 23.082 | 22.782 | 23.499 | 1.107 | 0.917 |
| 21 | GE MEDICAL SYSTEMS | Discovery CT750 HD | 18 | 19.049 | 11.702 | 8.745 | 15.694 | 15.306 | 15.694 | 0.919 | 0.778 |
| 22 | GE MEDICAL SYSTEMS | Discovery CT750 HD | 15 | 17.733 | 11.834 | 8.297 | 19.304 | 19.054 | 19.480 | 1.188 | 0.963 |
| 23 | GE MEDICAL SYSTEMS | Discovery CT750 HD | 32 | 20.366 | 12.305 | 10.135 | 17.147 | 16.937 | 16.946 | 1.294 | 1.121 |
| 24 | GE MEDICAL SYSTEMS | Discovery CT750 HD | 10 | 15.563 | 10.482 | 7.260 | 14.523 | 14.282 | 14.442 | 1.162 | 0.961 |
| 25 | GE MEDICAL SYSTEMS | Discovery CT750 HD | 21 | 18.791 | 12.272 | 8.919 | 20.484 | 20.212 | 20.603 | 1.009 | 0.857 |
| 26 | GE MEDICAL SYSTEMS | Discovery CT750 HD | 28 | 20.610 | 12.430 | 9.593 | 17.006 | 16.612 | 16.775 | 1.138 | 0.997 |
| 27 | GE MEDICAL SYSTEMS | Discovery CT750 HD | 21 | 15.728 | 10.359 | 7.432 | 18.509 | 18.542 | 18.722 | 1.504 | 1.181 |
| 28 | GE MEDICAL SYSTEMS | Discovery CT750 HD | 18 | 15.873 | 10.765 | 7.563 | 18.302 | 18.096 | 18.295 | 1.322 | 1.130 |
| 29 | GE MEDICAL SYSTEMS | Discovery CT750 HD | 21 | 19.285 | 12.577 | 9.193 | 15.250 | 15.015 | 15.186 | 0.873 | 0.742 |
| 30 | GE MEDICAL SYSTEMS | Discovery CT750 HD | 15 | 20.688 | 12.590 | 9.735 | 18.047 | 17.944 | 17.994 | 0.942 | 0.844 |
| 31 | Philips | Ingenuity CT | 76 | 15.579 | 10.787 | 7.709 | 17.349 | 16.393 | 16.156 | 1.123 | 1.060 |
| 32 | Philips | Ingenuity CT | 17 | 20.643 | 14.004 | 9.818 | 20.757 | 19.812 | 20.671 | 0.997 | 0.907 |
| 33 | Philips | Ingenuity CT | 21 | 17.263 | 11.941 | 8.496 | 18.950 | 18.521 | 19.104 | 0.752 | 0.704 |
| 34 | Philips | Ingenuity CT | 20 | 14.329 | 9.868 | 6.875 | 14.778 | 14.259 | 14.443 | 0.774 | 0.677 |
| 35 | Philips | Ingenuity CT | 28 | 17.922 | 12.528 | 8.552 | 22.697 | 22.091 | 23.113 | 1.367 | 1.283 |
| 36 | Philips | Ingenuity CT | 21 | 16.008 | 10.876 | 7.865 | 16.635 | 16.270 | 16.896 | 0.643 | 0.592 |
| 37 | Philips | Ingenuity CT | 29 | 16.653 | 11.481 | 8.212 | 19.452 | 19.043 | 19.592 | 0.809 | 0.737 |
| 38 | Philips | Ingenuity CT | 34 | 17.523 | 12.110 | 8.489 | 20.993 | 20.158 | 20.518 | 1.175 | 1.134 |
| 39 | Philips | Ingenuity CT | 25 | 17.070 | 11.639 | 8.254 | 18.975 | 18.243 | 18.674 | 0.894 | 0.815 |
| 40 | Philips | Ingenuity CT | 39 | 19.984 | 13.588 | 9.541 | 22.955 | 22.068 | 23.062 | 1.077 | 0.958 |
| 41 | Philips | Ingenuity CT | 31 | 17.537 | 12.094 | 8.431 | 15.405 | 14.931 | 15.274 | 0.787 | 0.721 |
| 42 | Philips | Ingenuity CT | 30 | 16.782 | 11.407 | 8.240 | 18.342 | 17.676 | 18.265 | 0.851 | 0.767 |
| 43 | Philips | Ingenuity CT | 36 | 15.817 | 10.745 | 7.791 | 16.498 | 15.735 | 16.079 | 0.979 | 0.917 |
| 44 | Philips | Ingenuity CT | 32 | 16.664 | 11.532 | 8.112 | 23.320 | 22.858 | 23.761 | 0.995 | 0.906 |
| 45 | Philips | Ingenuity CT | 21 | 14.797 | 10.089 | 7.223 | 17.293 | 16.441 | 16.543 | 1.035 | 0.959 |
| 46 | Philips | Ingenuity CT | 34 | 17.277 | 11.827 | 8.547 | 25.353 | 24.729 | 25.566 | 0.923 | 0.818 |
| 47 | Philips | Ingenuity CT | 30 | 17.335 | 11.977 | 8.470 | 20.260 | 19.733 | 20.175 | 0.892 | 0.837 |
| 48 | Philips | Ingenuity CT | 29 | 19.435 | 13.353 | 9.456 | 27.958 | 26.995 | 27.982 | 0.981 | 0.899 |
| 49 | Philips | Ingenuity CT | 29 | 17.567 | 12.257 | 8.573 | 22.540 | 21.826 | 22.562 | 0.995 | 0.887 |
| 50 | Philips | Ingenuity CT | 30 | 17.545 | 12.039 | 8.414 | 19.583 | 18.917 | 19.500 | 0.998 | 0.897 |
| 51 | Philips | Ingenuity CT | 33 | 17.397 | 12.060 | 8.195 | 17.805 | 17.299 | 17.784 | 0.964 | 0.901 |
| 52 | Philips | Ingenuity CT | 33 | 18.222 | 12.632 | 8.616 | 21.661 | 21.258 | 22.182 | 0.814 | 0.766 |
| 53 | Philips | Ingenuity CT | 34 | 19.086 | 13.039 | 9.350 | 19.383 | 18.554 | 19.191 | 0.904 | 0.829 |
| 54 | Philips | Ingenuity CT | 32 | 17.496 | 12.101 | 8.406 | 15.994 | 15.363 | 15.395 | 1.058 | 1.032 |
| 55 | Philips | Ingenuity CT | 32 | 18.393 | 12.700 | 8.816 | 21.362 | 20.719 | 21.573 | 0.947 | 0.836 |
| 56 | Philips | Ingenuity CT | 36 | 19.612 | 13.305 | 9.733 | 20.737 | 20.142 | 20.672 | 0.975 | 0.948 |
| 57 | Philips | Ingenuity CT | 30 | 20.557 | 14.168 | 9.809 | 22.137 | 21.589 | 22.590 | 0.849 | 0.796 |
| 58 | Philips | Ingenuity CT | 16 | 18.185 | 12.379 | 8.604 | 21.186 | 20.688 | 21.359 | 0.900 | 0.850 |
| 59 | Philips | Ingenuity CT | 36 | 18.703 | 12.967 | 8.699 | 19.187 | 18.708 | 19.323 | 0.877 | 0.839 |
| 60 | Philips | Ingenuity CT | 29 | 15.253 | 10.525 | 7.438 | 20.344 | 19.888 | 20.442 | 0.841 | 0.732 |
| 61 | SIEMENS | SOMATOM Definition Flash | 23 | 17.313 | 13.328 | 8.543 | 20.532 | 20.040 | 20.693 | 0.867 | 0.676 |
| 62 | SIEMENS | SOMATOM Definition Flash | 52 | 22.737 | 17.274 | 11.265 | 18.503 | 17.782 | 17.590 | 0.942 | 0.934 |
| 63 | SIEMENS | SOMATOM Definition Flash | 24 | 15.798 | 12.098 | 7.567 | 18.259 | 18.066 | 18.511 | 1.004 | 0.663 |
| 64 | SIEMENS | SOMATOM Definition Flash | 27 | 14.922 | 11.476 | 7.332 | 16.131 | 15.867 | 16.106 | 0.835 | 0.632 |
| 65 | SIEMENS | SOMATOM Definition Flash | 26 | 15.226 | 11.653 | 7.273 | 19.905 | 19.747 | 20.258 | 0.832 | 0.603 |
| 66 | SIEMENS | SOMATOM Definition Flash | 16 | 14.153 | 10.829 | 6.873 | 12.572 | 12.159 | 12.190 | 0.871 | 0.752 |
| 67 | SIEMENS | SOMATOM Definition Flash | 26 | 18.295 | 14.059 | 8.937 | 23.757 | 23.118 | 23.720 | 0.929 | 0.739 |
| 68 | SIEMENS | SOMATOM Definition Flash | 34 | 21.542 | 16.393 | 10.768 | 19.718 | 19.056 | 19.244 | 0.837 | 0.771 |
| 69 | SIEMENS | SOMATOM Definition Flash | 32 | 19.266 | 14.771 | 9.473 | 22.028 | 21.201 | 21.309 | 1.045 | 0.923 |
| 70 | SIEMENS | SOMATOM Definition Flash | 25 | 15.974 | 12.180 | 7.794 | 20.096 | 19.700 | 20.257 | 1.303 | 1.047 |
| 71 | SIEMENS | SOMATOM Definition Flash | 15 | 14.570 | 11.122 | 7.224 | 16.793 | 16.331 | 16.894 | 1.155 | 0.906 |
| 72 | SIEMENS | SOMATOM Definition Flash | 26 | 19.991 | 15.184 | 9.661 | 22.877 | 22.540 | 23.450 | 0.984 | 0.822 |
| 73 | SIEMENS | SOMATOM Definition Flash | 40 | 19.163 | 14.606 | 9.301 | 22.452 | 21.871 | 22.484 | 1.020 | 0.846 |
| 74 | SIEMENS | SOMATOM Definition Flash | 35 | 18.926 | 14.455 | 9.266 | 24.779 | 24.312 | 25.046 | 0.949 | 0.777 |
| 75 | SIEMENS | SOMATOM Definition Flash | 22 | 16.123 | 12.323 | 7.681 | 19.415 | 19.153 | 19.692 | 0.907 | 0.729 |
| 76 | SIEMENS | SOMATOM Definition Flash | 31 | 13.213 | 10.101 | 6.844 | 19.475 | 19.223 | 19.629 | 1.164 | 0.897 |
| 77 | SIEMENS | SOMATOM Definition Flash | 19 | 16.026 | 12.261 | 7.818 | 21.852 | 21.397 | 21.961 | 1.194 | 0.903 |
| 78 | SIEMENS | SOMATOM Definition Flash | 35 | 19.707 | 15.047 | 9.683 | 20.398 | 19.785 | 20.145 | 0.994 | 0.879 |
| 79 | SIEMENS | SOMATOM Definition Flash | 32 | 16.675 | 12.689 | 7.916 | 14.506 | 14.070 | 14.198 | 0.819 | 0.730 |
| 80 | SIEMENS | SOMATOM Definition Flash | 19 | 16.078 | 12.297 | 7.882 | 21.852 | 21.397 | 21.961 | 1.194 | 0.903 |
| 81 | SIEMENS | SOMATOM Definition Flash | 27 | 19.227 | 14.612 | 9.387 | 22.510 | 22.246 | 22.999 | 1.303 | 0.948 |
| 82 | SIEMENS | SOMATOM Definition Flash | 42 | 19.418 | 14.774 | 9.649 | 21.196 | 20.830 | 21.429 | 1.261 | 1.033 |
| 83 | SIEMENS | SOMATOM Definition Flash | 23 | 16.488 | 12.585 | 8.067 | 14.284 | 13.738 | 13.711 | 1.040 | 0.928 |
| 84 | SIEMENS | SOMATOM Definition Flash | 23 | 24.064 | 18.257 | 11.966 | 16.233 | 15.855 | 16.038 | 0.915 | 0.830 |
| 85 | SIEMENS | SOMATOM Definition Flash | 45 | 22.538 | 17.111 | 11.418 | 24.755 | 24.045 | 25.101 | 1.110 | 0.937 |
| 86 | SIEMENS | SOMATOM Definition Flash | 34 | 15.559 | 12.075 | 7.176 | 18.210 | 17.740 | 17.789 | 0.797 | 0.603 |
| 87 | SIEMENS | SOMATOM Definition Flash | 39 | 20.473 | 15.541 | 9.996 | 25.312 | 24.896 | 25.845 | 0.992 | 0.800 |
| 88 | SIEMENS | SOMATOM Definition Flash | 30 | 16.626 | 12.818 | 8.100 | 17.347 | 16.789 | 16.769 | 0.865 | 0.749 |
| 89 | SIEMENS | SOMATOM Definition Flash | 21 | 20.288 | 15.465 | 10.097 | 28.248 | 27.761 | 28.895 | 1.062 | 0.766 |
| 90 | SIEMENS | SOMATOM Definition Flash | 30 | 18.490 | 14.063 | 8.909 | 17.008 | 16.444 | 16.992 | 0.950 | 0.757 |
| 91 | TOSHIBA | Aquilion ONE | 25 | 29.045 | 14.666 | 14.633 | 22.197 | 21.030 | 21.395 | 1.283 | 1.124 |
| 92 | TOSHIBA | Aquilion ONE | 23 | 25.359 | 13.838 | 12.605 | 21.091 | 20.166 | 20.893 | 1.275 | 1.047 |
| 93 | TOSHIBA | Aquilion ONE | 27 | 28.973 | 13.446 | 14.429 | 21.212 | 20.600 | 21.188 | 0.968 | 0.856 |
| 94 | TOSHIBA | Aquilion ONE | 21 | 27.133 | 14.265 | 13.468 | 22.461 | 21.343 | 22.444 | 1.034 | 0.837 |
| 95 | TOSHIBA | Aquilion ONE | 24 | 28.169 | 15.549 | 14.122 | 23.222 | 22.665 | 23.822 | 1.016 | 0.854 |
| 96 | TOSHIBA | Aquilion ONE | 20 | 22.346 | 13.210 | 11.204 | 18.612 | 17.836 | 18.270 | 0.940 | 0.771 |
| 97 | TOSHIBA | Aquilion ONE | 15 | 20.139 | 12.135 | 9.811 | 11.810 | 11.388 | 11.321 | 0.675 | 0.611 |
| 98 | TOSHIBA | Aquilion ONE | 19 | 29.339 | 14.843 | 14.806 | 24.416 | 23.419 | 24.870 | 0.894 | 0.738 |
| 99 | TOSHIBA | Aquilion ONE | 33 | 28.467 | 14.395 | 14.347 | 17.031 | 16.105 | 15.906 | 0.930 | 0.859 |
| 100 | TOSHIBA | Aquilion ONE | 20 | 23.620 | 13.823 | 11.636 | 21.100 | 20.180 | 21.120 | 1.125 | 0.912 |
| 101 | TOSHIBA | Aquilion ONE | 29 | 23.485 | 13.145 | 11.501 | 14.559 | 14.019 | 13.979 | 0.920 | 0.848 |
| 102 | TOSHIBA | Aquilion ONE | 25 | 27.577 | 14.306 | 13.970 | 21.657 | 20.643 | 21.550 | 0.998 | 0.833 |
| 103 | TOSHIBA | Aquilion ONE | 24 | 26.726 | 14.339 | 13.289 | 19.711 | 18.711 | 18.801 | 1.102 | 0.999 |
| 104 | TOSHIBA | Aquilion ONE | 26 | 29.028 | 14.221 | 14.369 | 26.101 | 24.857 | 26.031 | 1.247 | 1.070 |
| 105 | TOSHIBA | Aquilion ONE | 27 | 27.879 | 14.494 | 14.130 | 18.261 | 17.435 | 17.776 | 1.158 | 1.013 |
| 106 | TOSHIBA | Aquilion ONE | 20 | 25.811 | 13.663 | 12.981 | 17.822 | 17.111 | 17.689 | 0.843 | 0.716 |
| 107 | TOSHIBA | Aquilion ONE | 20 | 25.799 | 13.640 | 12.754 | 19.645 | 18.546 | 19.299 | 1.045 | 0.901 |
| 108 | TOSHIBA | Aquilion ONE | 17 | 22.767 | 13.217 | 11.134 | 15.791 | 15.174 | 15.466 | 1.122 | 0.944 |
| 109 | TOSHIBA | Aquilion ONE | 33 | 29.986 | 15.056 | 15.270 | 25.239 | 24.087 | 25.354 | 1.181 | 0.978 |
| 110 | TOSHIBA | Aquilion ONE | 30 | 27.740 | 14.209 | 13.843 | 20.050 | 19.295 | 19.544 | 1.127 | 1.023 |
| 111 | TOSHIBA | Aquilion ONE | 12 | 23.736 | 13.591 | 11.832 | 16.495 | 15.716 | 16.160 | 0.817 | 0.673 |
| 112 | TOSHIBA | Aquilion ONE | 16 | 26.259 | 14.196 | 13.406 | 23.047 | 22.123 | 23.215 | 0.927 | 0.779 |
| 113 | TOSHIBA | Aquilion ONE | 28 | 29.342 | 15.518 | 15.189 | 20.347 | 19.642 | 20.195 | 1.227 | 1.120 |
| 114 | TOSHIBA | Aquilion ONE | 20 | 25.880 | 14.029 | 13.167 | 23.245 | 22.152 | 22.931 | 1.059 | 0.917 |
| 115 | TOSHIBA | Aquilion ONE | 25 | 29.905 | 15.855 | 15.169 | 20.429 | 19.699 | 20.304 | 1.175 | 1.073 |
| 116 | TOSHIBA | Aquilion ONE | 24 | 25.033 | 13.128 | 12.182 | 19.368 | 18.761 | 19.443 | 1.216 | 1.047 |
| 117 | TOSHIBA | Aquilion ONE | 21 | 26.671 | 14.265 | 13.409 | 23.896 | 22.930 | 24.280 | 1.039 | 0.827 |
| 118 | TOSHIBA | Aquilion ONE | 23 | 25.630 | 14.054 | 12.693 | 20.628 | 19.645 | 20.366 | 1.186 | 0.990 |
| 119 | TOSHIBA | Aquilion ONE | 14 | 22.753 | 13.419 | 11.390 | 18.668 | 18.034 | 18.729 | 0.840 | 0.660 |
| 120 | TOSHIBA | Aquilion ONE | 21 | 29.421 | 14.479 | 14.928 | 20.540 | 19.660 | 20.304 | 1.029 | 0.896 |
